# Electrocorticographic Activation within Human Auditory Cortex during Dialog-Based Language and Cognitive Testing

**DOI:** 10.3389/fnhum.2016.00202

**Published:** 2016-05-04

**Authors:** Kirill V. Nourski, Mitchell Steinschneider, Ariane E. Rhone

**Affiliations:** ^1^Human Brain Research Laboratory, Department of Neurosurgery, The University of Iowa, Iowa CityIA, USA; ^2^Departments of Neurology and Neuroscience, Albert Einstein College of Medicine, BronxNY, USA

**Keywords:** Heschl’s gyrus, high gamma, Mini-mental state examination, speech, superior temporal gyrus

## Abstract

Current models of cortical speech and language processing include multiple regions within the temporal lobe of both hemispheres. Human communication, by necessity, involves complex interactions between regions subserving speech and language processing with those involved in more general cognitive functions. To assess these interactions, we utilized an ecologically salient conversation-based approach. This approach mandates that we first clarify activity patterns at the earliest stages of cortical speech processing. Therefore, we examined high gamma (70–150 Hz) responses within the electrocorticogram (ECoG) recorded simultaneously from Heschl’s gyrus (HG) and lateral surface of the superior temporal gyrus (STG). Subjects were neurosurgical patients undergoing evaluation for treatment of medically intractable epilepsy. They performed an expanded version of the Mini-mental state examination (MMSE), which included additional spelling, naming, and memory-based tasks. ECoG was recorded from HG and the STG using multicontact depth and subdural electrode arrays, respectively. Differences in high gamma activity during listening to the interviewer and the subject’s self-generated verbal responses were quantified for each recording site and across sites within HG and STG. The expanded MMSE produced widespread activation in auditory cortex of both hemispheres. No significant difference was found between activity during listening to the interviewer’s questions and the subject’s answers in posteromedial HG (auditory core cortex). A different pattern was observed throughout anterolateral HG and posterior and middle portions of lateral STG (non-core auditory cortical areas), where activity was significantly greater during listening compared to speaking. No systematic task-specific differences in the degree of suppression during speaking relative to listening were found in posterior and middle STG. Individual sites could, however, exhibit task-related variability in the degree of suppression during speaking compared to listening. The current study demonstrates that ECoG recordings can be acquired in time-efficient dialog-based paradigms, permitting examination of language and cognition in an ecologically salient manner. The results obtained from auditory cortex serve as a foundation for future studies addressing patterns of activity beyond auditory cortex that subserve human communication.

## Introduction

Intracranial recordings in humans have permitted evaluation of speech and language processing with unprecedented temporal and spatial resolution (e.g., Leonard and Chang, 2014; Nourski and Howard, 2015). Most of these intracranial studies have focused on neural activity on the lateral surface of the STG (e.g., Crone et al., 2001; Steinschneider et al., 2011; Mesgarani et al., 2014). For instance, Mesgarani et al. (2014) have demonstrated a role for the posterior lateral STG of the dominant hemisphere in acoustic-to-phonetic transformations of speech. Less explored are regions of auditory and auditory-related cortex envisioned to encode ever more complex features of speech and language. For instance, cortex within the superior temporal sulcus and middle temporal gyrus is critical for phonological and lexical-semantic processing, respectively (Binder et al., 2000; Obleser et al., 2008; Hickok, 2009; Leaver and Rauschecker, 2010). Furthermore, regions of the brain involved in cognitive processes such as attention, working memory, and declarative memory must by necessity interface with regions of the brain more directly involved in speech processing.

The opportunity to simultaneously explore multiple brain regions involved in speech and language is provided by the extensive electrode coverage in epilepsy patients undergoing chronic invasive monitoring. However, paradigms investigating complex speech and language functions must take into account that these studies are being carried out in patients in a hospital setting with the primary goal being remediation of their seizure disorders. These considerations mandate that these studies be time-efficient and performed with the recognition that prolonged experimental sessions often engender excessive patient fatigue and potentially lead to unwillingness to pursue further participation in research activities.

In this study, we initiated a conversation-based paradigm that incorporates multiple speech, language, and cognitive functions in a time-efficient manner. We hypothesized that such a paradigm would be a more ecologically salient means to study these complex functions than traditionally used trial-based protocols (e.g., Steinschneider et al., 2014; Nourski et al., 2015a). A conversation, by its very nature, will engage a wide array of auditory, speech, and language areas and interface with regions engaged in higher cognitive functions. This conversation-based approach has been shown to be an effective means for exploring the roles of human auditory and auditory-related cortex within the setting of clinically necessitated intracranial recordings (Creutzfeldt et al., 1989; Derix et al., 2012, 2014; see also Dastjerdi et al., 2013).

For these reasons, we utilized the MMSE, which is a commonly used tool to screen for language and cognitive impairments associated with dementia (Finney et al., 2016). It examines a range of functions, including orientation to time and place, immediate and delayed recall, attention, naming, repetition, and following multi-step commands (Folstein et al., 1975). However, it has been recently noted that the MMSE lacks sufficient sensitivity and specificity in predicting dementia and thus should not be used as a standalone clinical test for screening of language and cognitive deficits (Arevalo-Rodriguez et al., 2015). Therefore, we have implemented additional tasks for a more comprehensive assay of the cortical regions involved in higher language and cognitive functions. These tasks included digit span, spelling, rhyming, abstract naming, verbal analogies, sentence comprehension, fund of knowledge, and identification of favorite items. The expanded paradigm is highly time-efficient and is typically completed within approximately 15 min.

Despite its potential utility, this conversation-based experimental paradigm presents several challenges when analyzing task-related cortical activity using ECoG (Nourski and Howard, 2015). Conventional trial-based paradigms typically rely on analyzing activity that is time-locked to particular events by averaging across multiple instances of these events. These analyses typically focus on low-frequency local field potentials or activity in the high gamma (70–150 Hz) ECoG frequency range (e.g., Crone et al., 2001; Nourski et al., 2015b). Studies examining high gamma ECoG often do so by referencing event-related activity to a pre-defined local baseline (ERBP). However, a conversation-based paradigm offers neither repetition of the same event, nor a stable local baseline. To deal with these issues in the present study, cortical high gamma activity was normalized relative to mean power over the entire duration of the recording, and then averaged across all utterances, done separately for the interviewer’s and the subject’s speech.

Due to the challenges of this new method, we initiated our investigation in lower auditory cortical areas with relatively well-described basic response properties (e.g., Brugge et al., 2008; Leonard and Chang, 2014; Mesgarani et al., 2014; Nourski et al., 2014a,b). Specifically, we focused our initial investigation on neural activity generated within the auditory cortex located in HG and on the lateral surface of the STG. These regions incorporate portions of auditory core, belt and parabelt cortex (e.g., Hackett et al., 2001; Brugge et al., 2008; Nourski et al., 2014a; Hackett, 2015). Analysis was restricted to activity in the high gamma frequency range, which has been shown to be useful in defining the basic physiological response properties of these cortical regions (e.g., Crone et al., 2001; Brugge et al., 2009; Steinschneider et al., 2011). Identification of high gamma response patterns within auditory cortex is a necessary prerequisite for clarifying patterns of activity at higher stages of cortical speech and language processing.

The posteromedial portion of HG has been consistently identified as part of core auditory cortex (e.g., Liegeois-Chauvel et al., 1991; Brugge et al., 2008; Nourski et al., 2014a). Electrophysiological studies have demonstrated that this brain region is strongly activated by a wide range of simple and complex sound stimuli. It is unclear, however, whether activity would be different for sounds generated by the interviewer versus sounds self-initiated by the subject. Suppression of activity during self-initiated speech has been demonstrated in both non-human primates (Müller-Preuss and Ploog, 1981; Eliades and Wang, 2003, 2005) and humans (Creutzfeldt et al., 1989; Houde et al., 2002; Greenlee et al., 2011). While suppression has been demonstrated within auditory core cortex in the non-human primate (Eliades and Wang, 2003, 2005), it has not been demonstrated in the human (Greenlee et al., 2014; Behroozmand et al., 2016). We therefore examined whether activity in posteromedial HG would be modulated by speaker during a conversation. A similar logic applies to whether suppression of activity elicited by self-initiated speech would occur within non-core cortex in anterolateral HG.

Auditory cortex on the lateral STG has been shown to be modulated by speech phonetic features, attention and task demands, and self-initiated vocalization (e.g., Chang et al., 2011; Greenlee et al., 2011; Mesgarani and Chang, 2012; Mesgarani et al., 2014; Steinschneider et al., 2014). While these studies have been performed in well-structured and controlled settings, it remains to be seen whether these effects can be reliably identified within the ecologically relevant context of a conversation-based paradigm.

Thus, in the present study, we examined modulation of activity elicited when listening and speaking during performance of the expanded MMSE within four ROIs: posteromedial HG, anterolateral HG, posterior STG, and middle STG. Decoding of complex and abstract features of speech occurs in more anterior regions of the temporal lobe (Hickok and Poeppel, 2007; Hickok, 2009). The TTS provides an anatomical landmark that may be useful for demarcating posterior from middle portions of STG. We therefore reasoned that modulation of activity due to self-vocalization might vary between these two regions of the STG. We further examined whether activity was modulated by the multiple tasks incorporated in our expanded version of the MMSE.

## Materials and Methods

### Subjects

Experimental subjects were six neurosurgical patients (three female, three male, age 21–51 years old, median age 33 years old) diagnosed with medically refractory epilepsy undergoing chronic invasive ECoG monitoring to identify potentially resectable temporal lobe seizure foci. Demographic data for each subject are presented in **Table 1**. Research protocols were approved by the University of Iowa Institutional Review Board and the National Institutes of Health. Written informed consent was obtained from all subjects. Research participation did not interfere with acquisition of clinically required data, and subjects could rescind consent at any time without interrupting their clinical evaluation.

**Table 1:** Subject demographics.

Subject^1^	Age	Sex	Handedness	Language dominance	MMSE score^2^
R288	21	M	R	L	26/27
L292	50	F	L	R	26/27
R294	35	M	R	L	26/27
L307	29	M	R	L	24/27
R316	31	F	R	L	24/27
R320	51	F	R	L	24/27

*^1^Letter prefix of the subject code denotes the side of electrode implantation (L, left; R, right). ^2^Three of the standard MMSE tasks (reading, writing, and copying) were not included in the current protocol, therefore the maximum MMSE score was 27 rather than 30.*

All subjects underwent audiometric evaluation before the study, and none was found to have hearing deficits that should impact the findings presented in this study. All subjects had pure-tone thresholds within 25 dB HL between 250 Hz and 4 kHz, with the exception of subject L307, who had a mild (40 dB HL) notch at 4 kHz in the right ear only. All subjects were native English speakers. Intracranial recordings revealed that auditory cortical areas within the four ROI in HG and on STG were not epileptic foci in any subject.

### Procedure

Experiments were carried out in a dedicated electrically shielded suite in The University of Iowa Clinical Research Unit. The subjects were comfortably reclining in a hospital bed or an armchair while performing the MMSE (Folstein et al., 1975). In subjects L307, R316, and R320, testing was expanded beyond the MMSE to include other tasks (digit span, spelling, rhyming, naming, verbal analogies, sentence comprehension, and fund of knowledge). These subjects were also asked to identify favorite items (e.g., favorite food or movie; Supplementary Table 1).

All subjects had comparable performance in aspects of the MMSE, with “Delayed Verbal Recall” being the only section where all subjects had difficulty (see **Table 1**). Three subjects failed to recall one out of three words, while three others could not recall any of the three words. It should be noted that the interviewer did not specifically emphasize that the subjects would be asked to recall the three words later in the test. Overall, the subjects’ successful performance on the exam indicated that neural activity was not biased by cognitive deficits specifically revealed by the MMSE.

### Recordings

Electrocorticography recordings were simultaneously made from HG and the lateral cortical surface using multicontact depth and subdural grid electrodes, respectively. Details of electrode implantation, recording, and analysis of high gamma cortical activity have been previously described in depth (Howard et al., 1996, 2000; Reddy et al., 2010; Nourski et al., 2013; Nourski and Howard, 2015). All electrode arrays were placed solely on the basis of clinical requirements, and were part of a more extensive set of recording arrays meant to identify seizure foci. Electrodes remained in place under the direction of the patients’ treating neurologists.

Depth electrode arrays (eight macro contacts, spaced 5 mm apart) were implanted in each subject stereotactically into HG, along its anterolateral-to-posteromedial axis. The approach used at The University of Iowa is modeled in part after the well-established stereo-EEG techniques developed and used widely in epilepsy centers in Europe. The technique involves implantation of electrodes within the superior temporal plane in order to provide broad coverage of the suspected seizure focus. With this strategy, electrodes are implanted in the superior temporal plane regardless of whether a patient with suspected temporal lobe seizures describes auditory auras (Munari, 1987; Bartolomei et al., 1999, 2008; Maillard et al., 2004; Gavaret et al., 2006; McGonigal et al., 2007). Review of all patients who had been implanted with depth electrodes in the superior temporal plane within the last 3 years revealed the strong clinical utility of the ECoG data provided by these electrodes in clinical decision making with regard to the extent of surgical resections (data available upon request).

Subdural grid arrays were implanted over the lateral surface of the cerebral hemisphere, including the auditory cortex on the lateral STG. The grid arrays consisted of platinum–iridium disk electrodes (2.3 mm exposed diameter) embedded in a silicon membrane. In subjects R288, L307, and R320 high density (5 mm center-to-center inter-electrode distance) research grids were used, with electrodes arranged in an 8 × 12 grid, yielding a 3.5 cm × 5.5 cm array of 96 contacts. In subject R316, a 32-contact clinical grid (4 × 8 array with a 10 mm inter-electrode distance) was used. In subjects L292 and R294, 16-contact clinical grids (2 × 8 array, 10 mm inter-electrode distance) were placed over the lateral surface of the STG. In all subjects, a subgaleal contact was used as a reference.

As with the depth electrodes, decisions regarding what surface regions and to what extent should be covered, are driven exclusively by clinical considerations. High resolution research grids do not increase the risks of surgery or alter the area of cortex from which records are obtained. Also, the materials used to fabricate the arrays that are in contact with the brain surface are the same for research and clinical electrodes. Information about electrodes modified for research purposes was conveyed to each patient prior to surgery.

Subjects underwent whole-brain high-resolution T1-weighted structural MRI scans (resolution 0.78 mm × 0.78 mm, slice thickness 1.0 mm) before electrode implantation. Two volumes were averaged to improve the signal-to-noise ratio of the MRI data sets and minimize the effects of movement artifact on image quality. After electrode implantation, subjects underwent thin-sliced volumetric computerized tomography scans (resolution 0.51 mm × 0.51 mm, slice thickness 1.0 mm).

Locations of recording sites were determined by co-registering pre- and post-implantation structural imaging data using a linear algorithm with six degrees of freedom (Jenkinson et al., 2002), aided by intraoperative photographs.

Data acquisition was controlled by a TDT RZ2 real-time processor (Tucker-Davis Technologies, Alachua, FL, USA). Collected ECoG data were amplified, filtered (0.7–800 Hz bandpass, 12 dB/octave rolloff), digitized at a sampling rate of 2034.5 Hz, and stored for subsequent oﬄine analysis. The conversation between the interviewer and subject was recorded simultaneously using an in-room Behringer ECM 8000 microphone (Behringer, Willich, Germany) and digitized at a sampling rate of 12207 Hz.

### Data Analysis

Utterances spoken by the interviewer and the subject were parsed using Praat software based upon specific phrases and natural breaks in the conversation, generally following a question–answer format. This method was chosen in order to compare activity elicited during listening versus speaking across ROIs. Average durations of utterances by the interviewer and the subjects parsed using this method were not significantly different (Wilcoxon rank sum test, **Table 2**). Voice fundamental frequency (*F*_0_) was estimated for each utterance using YIN fundamental frequency estimator (de Cheveigné and Kawahara, 2002). Two of the subjects (L292, R316) had median *F*_0_s significantly higher than the interviewer, one subject (L307) had significantly lower *F*_0_, while the other three subjects did not exhibit significant differences in *F*_0_ from the interviewer (Wilcoxon rank sum test; see **Table 2**).

**Table 2:** Acoustic properties of dialogues between the interviewer and each subject.

Subject	Utterance duration			*F*_0_		
	Median utterance duration (interviewer) (s)	Median utterance duration (subject) (s)	Interviewer vs. subject comparison *p*	Median *F*_0_ (interviewer) (Hz)	Median *F*_0_ (subject) (Hz)	Interviewer vs. subject comparison *p*
R288	1.934	1.457	0.205	127.7	142.9	0.305
L292	1.738	1.464	0.271	155.0	204.4	<0.001
R294	1.441	1.435	0.918	137.9	141.4	0.593
L307	1.709	1.440	0.815	131.2	120.7	<0.001
R316	1.622	1.139	0.142	139.8	205.2	<0.001
R320	1.309	1.168	0.477	163.7	181.2	0.711
All subjects	1.519	1.441				

Electrocorticography data obtained from each recording site were downsampled to 1000 Hz. To minimize contamination from power line noise, ECoG waveforms were de-noised using an adaptive notch filtering procedure (Nourski et al., 2013). Data analysis was performed using custom software written in the MATLAB Version 7.14 programming environment (MathWorks, Natick, MA, USA).

Analysis of cortical activity focused on the high gamma ECoG frequency band. High gamma power envelope was calculated for each recording site. ECoG waveforms were bandpass filtered between 70 and 150 Hz (300th order finite impulse response filter), followed by Hilbert envelope extraction and smoothing using a moving average filter with a span of 25 ms.

For quantitative analysis, high gamma ERBP was computed in all subjects as follows: power envelope waveforms were log-transformed, high-pass filtered (fourth order Butterworth filter, 0.1 Hz cutoff) to eliminate long-term baseline changes, and normalized to the mean power over the entire duration of the recording. ERBP was then averaged within time windows corresponding to each utterance (between 50 ms after the onset and 200 ms after the offset of each utterance), and averaged separately across all utterances spoken by the interviewer and the subject. This time window has been shown to capture the excitatory responses to speech, as well as suppression in high gamma activity during self-vocalization (see Greenlee et al., 2011). Supplementary Figure 1 demonstrates this window for high gamma activity elicited by all utterances in subjects L307 and R320. The analysis to establish the time window of interest was carried out in these two subjects because they had extensive coverage of the STG and were presented with the expanded MMSE questionnaire. On average, onset of activity began approximately 50 ms after the onset of the utterance, and persisted for approximately 200 ms following the offset of the utterance. It must be acknowledged that this approach limits the ability to assess the neural dynamics underlying the processing fine-grain spectrotemporal attributes within speech stimuli (cf. Mesgarani et al., 2014). However, the purpose of this paradigm is to characterize brain regions processing the utterances as a whole, thus promoting identification of neural dynamics related to specific language and cognitive tasks. Finally, activity during silent intervals between the interviewer’s questions and the subject’s verbal responses was averaged within time windows between 250 ms after the interviewer’s utterance offset and the onset of the next utterance. These time windows were then used for quantitative analysis of high gamma activity elicited during listening, speaking, and the intervening silence in all six subjects.

Previous studies have demonstrated that acoustically responsive cortex in HG and on STG comprises multiple fields, with posteromedial HG consistently interpreted as core auditory cortex. To approximate this complex multi-field functional organization, both HG and STG in each subject were subdivided into ROIs for quantitative analysis of high gamma activity recorded during the MMSE. Recording sites within HG were subdivided into posteromedial and anterolateral ROIs based on physiological criteria (Brugge et al., 2008, 2009). Specifically, recording sites were assigned to the posteromedial HG ROI if they exhibited phase-locked ECoG responses to 100 Hz click trains and averaged evoked potentials to these stimuli featured short-latency (<20 ms) components. Such response features are not present within anterolateral HG. Recording sites on the lateral surface of STG were subdivided into posterior and middle STG ROIs based on their location relative to the TTS, which is a continuation of Heschl’s sulcus onto the lateral surface of the STG. This anatomical demarcation is supported by previous work demonstrating that phonological processing primarily engaged areas of the STG posterior to the TTS (Hickok and Poeppel, 2007; Hickok, 2009).

Following the approach of Eliades and Wang (2003) and Greenlee et al. (2011), differences in high gamma activity between listening and speaking were first evaluated for each recording site using the SI metric:

$$\text{SI}=\frac{\;\gamma_{\text{listening}}\text{-}\gamma_{\text{speaking}}}{\;\gamma_{\text{listening}}\text{+}\gamma_{\text{speaking}}}$$

where γ_listening_ and γ_speaking_ are median high gamma power within the time windows corresponding to listening and speaking, respectively. For each ROI, SI values were compared to zero using Wilcoxon signed-rank tests.

The use of SI in this study differs from previous studies that compared auditory responses to self-initiated vocalizations with responses elicited by playback of the same utterances (e.g., Eliades and Wang, 2003; Greenlee et al., 2013). In contrast, the present study defined SI based on different speech material, specifically, comparisons between auditory responses elicited during listening to the interviewer and during verbally responding. The SI was used in a manner similar to a study that examined suppression of auditory activity on lateral STG during a repetition task (Flinker et al., 2010). Our study is novel in that it extends the findings of previous studies that used the same speech material to a conversational scenario.

Non-parametric statistical analysis was used for comparisons of high gamma ERBP between speaker conditions (interviewer vs. subject) and ROIs (posteromedial vs. anterolateral HG and posterior vs. middle STG). Wilcoxon rank sum test was used to compare average high gamma ERBP during listening to instructions of the interviewer and to the subject’s own verbal responses. Wilcoxon signed-rank test was used for ROI comparisons. Correction for multiple comparisons was done by controlling FDR (Benjamini and Hochberg, 1995) using the linear step-up procedure, as implemented in MATLAB Version 7.14 Bioinformatics Toolbox. Previous work has demonstrated the utility of this statistical approach when examining ECoG recorded during a conversation-based paradigm (Derix et al., 2012).

## Results

### Heschl’s Gyrus

**FIGURE 1:**
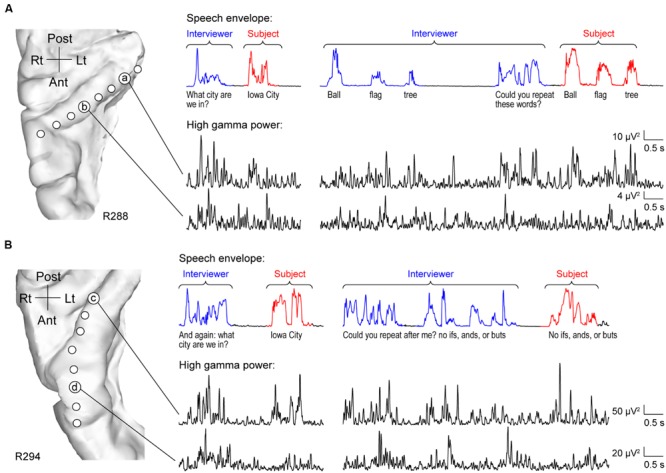
**Activity in HG during performance of MMSE. (A)** Exemplary data from subject R288. Left panel: MRI top-down view of right superior temporal plane showing the locations of recording contacts chronically implanted in HG. Right panel: speech envelopes of excerpts of the conversation, with interviewer’s and subject’s utterances highlighted in blue and red, respectively (top) and simultaneously recorded high gamma power from two representative sites in posteromedial and anterolateral HG (a and b, respectively; bottom). The transcript of the conversation is shown immediately below the speech envelopes. **(B)** Exemplary data from subject R294. Panels are arranged in the same way as in **(A)**.

As expected, HG was strongly activated by speech. However, activity was not uniform across its length. Two principal patterns of neural activity were identified that related to whether the utterances were the interviewer’s questions, or were self-generated by the subject in response to these questions. These two patterns were anatomically segregated along HG. Specifically, activity recorded from sites within posteromedial HG was characterized by robust increases in high gamma power when the subject was both listening and speaking. This pattern is exemplified by data from two subjects (R288 and R294) in **Figure 1** (sites ‘a’ and ‘c’). Increases in high gamma power were time-locked to the utterances of both the interviewer and subject. The second pattern was observed in anterolateral HG (sites ‘b’ and ‘d’ in **Figure 1**), wherein high gamma activity was generally of lower amplitude in response to self-initiated speech compared to listening.

**FIGURE 2:**
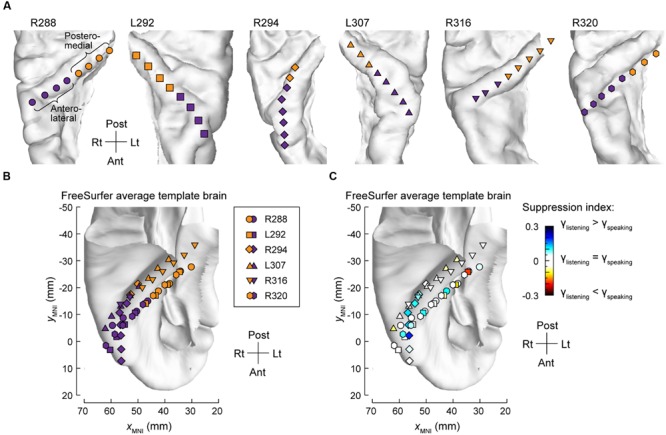
**Anatomical parcellation of HG and summary of SI analysis. (A)** MRI superior temporal plane top-down views showing the locations of recording contacts chronically implanted in HG in all subjects. Recording sites assigned to posteromedial and anterolateral ROIs as described in Section “Materials and Methods” are shown in orange and purple, respectively. **(B)** Locations of all posteromedial and anterolateral HG sites (orange and purple symbols, respectively) plotted in MNI coordinate space and projected onto the FreeSurfer average template brain. Different symbol shapes correspond to different subjects. **(C)** Color-coded SI values (threshold ± 0.05), plotted in MNI coordinate space and projected onto FreeSurfer average template brain. Different symbol shapes correspond to different subjects (as shown in the legend of panel **B**).

The differences between high gamma activity in posteromedial and anterolateral HG were quantified for all subjects on an utterance-by-utterance basis by comparing activity elicited during listening and self-vocalizations (**Figure 2**). Locations of the recording sites along HG in all six subjects are shown in **Figure 2A**. Recording sites are color-coded according to whether they were in posteromedial or anterolateral portions of HG as determined physiologically by responses to simple non-speech stimuli (see Materials and Methods). These locations, pooled across all subjects and transferred onto the right HG, are plotted in MNI coordinate space over the FreeSurfer average template brain in **Figure 2B**. Pooling anatomical data across subjects demonstrated that ROI demarcation based on physiological response properties in individual subjects translated into anatomically distinct regions within HG at the population level. This finding supports the reliability of the physiology-based operational definitions of posteromedial (core) and anterolateral HG (non-core) cortex as implemented in the present study.

Changes in high gamma activity during listening vs. speaking were quantified as SIs for each recording site across the entire conversation (see Materials and Methods). Recording sites in posteromedial HG were characterized by SIs that were not significantly different than zero (Wilcoxon signed-rank test *p* = 0.57), indicating a comparable degree of activation during listening and speaking (**Figure 2C**). In contrast, sites localized to the anterolateral portion of HG did exhibit positive SIs (Wilcoxon signed-rank test *p* < 0.005), corresponding to a greater degree of activation during listening versus speaking.

**FIGURE 3:**
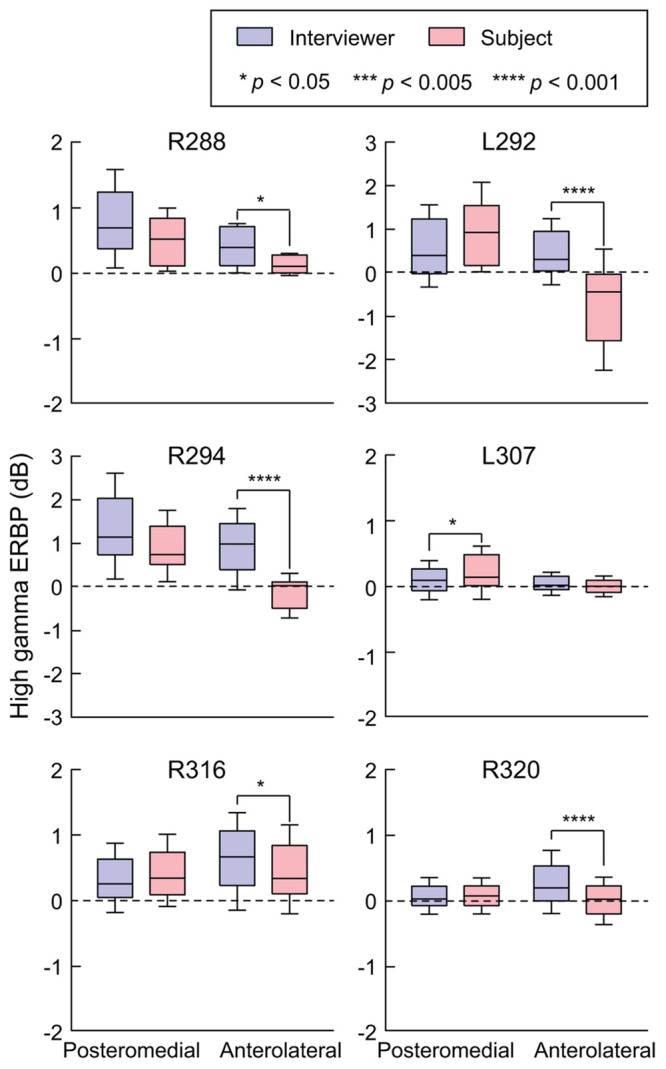
**Summary of HG ERBP analysis.** High gamma power was log-transformed and normalized to the mean power over the entire duration of the recording. Box plots show median, quartile, 10th and 90th percentile values of high gamma ERBP averaged over all interviewer’s and subject’s utterances (blue and red boxes, respectively) and recording sites within each ROI in each subject. Significance of ERBP differences was evaluated using Wilcoxon rank sum tests, followed by FDR correction.

Site-by-site analysis of SIs was effective in identifying differential patterns of speech-elicited activity along HG based on whether or not it was self-generated. This finding was confirmed by quantifying the differences between normalized high gamma activity (ERBP) measured during listening and speaking within the two HG ROIs (**Figure 3**). Utterance-by-utterance average high gamma power elicited during listening and self-initiated speech was calculated for each ROI in each subject. In posteromedial HG, activity elicited during listening and self-vocalization was of similar magnitude (Wilcoxon rank sum test, FDR-corrected, *p* > 0.05) in five out of six subjects. In the sixth subject (L307) activity was greater during self-vocalization (*p* < 0.05). In contrast, activity in anterolateral HG was greater while listening in five out of six subjects (*p* < 0.05). In the sixth subject (L307), responses were not significantly different.

In summary, there was a significant change in high gamma activity patterns along HG, wherein its posteromedial portion exhibited robust responses to conversational speech regardless of the speaker, while its anterolateral aspect responded more strongly during listening.

### Superior Temporal Gyrus

**FIGURE 4:**
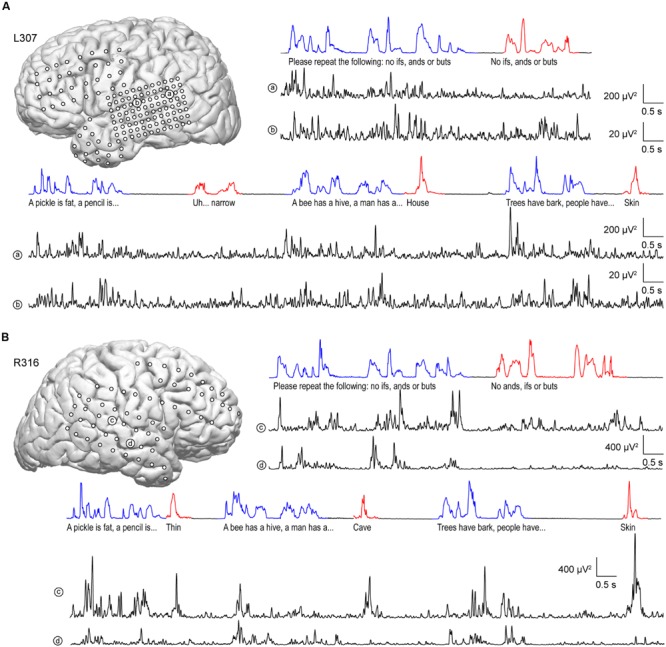
**Activity in STG during performance of the expanded MMSE. (A)** Data from left (language-dominant) STG (subject L307). Inset: MRI view of left hemisphere showing the locations of chronically implanted subdural electrodes. Speech envelopes of two excerpts of the conversation (interviewer’s and subject’s utterances highlighted in blue and red, respectively) are shown along with simultaneously recorded high gamma power from two representative sites in posterior and middle STG (a and b, respectively) underneath. **(B)** Data from right (language non-dominant) STG (subject R316). Panels are arranged in the same way as in **(A)**.

Similar to anterolateral HG, there was significant suppression of high gamma activity in response to self-initiated speech relative to listening on most sites along STG, as exemplified in **Figure 4**. In the language-dominant hemisphere of subject L307, site ‘a’ exhibited marked suppression of high gamma activity when the subject was speaking regardless of the task (**Figure 4A**). On a more anterior site ‘b,’ this suppression was more nuanced, with greater suppression occurring during the Verbal Analogies task compared to the Repetition task. The latter finding was comparable in the Immediate Recall task of the MMSE. Similar response patterns were observed in the non-language dominant hemisphere, exemplified by sites ‘c’ and ‘d’ in subject R316 (**Figure 4B**). In this subject, site ‘c’ again showed a more nuanced pattern of activity. In contrast to site ‘b,’ responses to the subject’s own speech were comparable to those when listening during the Verbal Analogies task, whereas suppression during speaking was evident during the Repetition task. A more anterior site ‘d’ showed a uniform pattern of marked suppression of activity when speaking, similar to site ‘a’ of subject L307.

**FIGURE 5:**
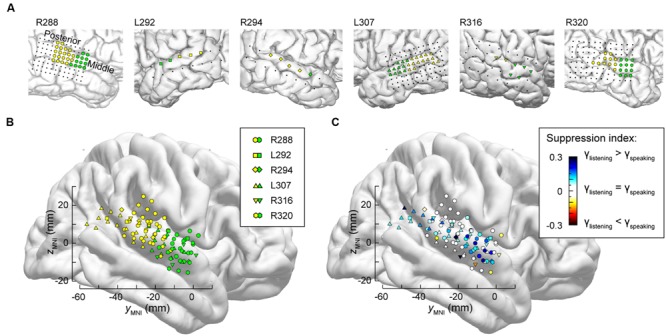
**Anatomical parcellation of STG and summary of SI analysis. (A)** MRI views showing the locations of recording contacts chronically implanted over STG in all subjects. Recording sites assigned to posterior and middle STG ROIs as described in text are shown in yellow and green, respectively. Recording sites on the subdural temporal grid arrays that were excluded from analyses on anatomical grounds are depicted as black dots. **(B)** Locations of all posterior and middle STG sites (yellow and green symbols, respectively) plotted in MNI coordinate space and projected onto FreeSurfer average template brain. Different symbol shapes correspond to different subjects. **(C)** Color-coded SI values (threshold ± 0.05), plotted in MNI coordinate space and projected onto FreeSurfer average template brain. Different symbol shapes correspond to different subjects.

It is likely that lateral STG contains multiple functional fields along its posterior-to-anterior axis (e.g., Hickok, 2009; Rauschecker and Scott, 2009). Accordingly, the distribution of electrodes along STG was examined to determine whether there were differences in suppression in posterior vs. middle portions of the STG. As physiological criteria currently do not provide a reliable means of identifying spatially distinct functional fields along the STG, anatomical criteria were used instead, based on the location of electrodes relative to the TTS (**Figure 5A**).

Superior temporal gyrus recording sites were then pooled across all six subjects and plotted in MNI coordinate space over the right hemisphere of the FreeSurfer average template brain (**Figure 5B**). In parallel with the evaluation of HG parcellation (cf. **Figure 2B**), there was concordance between STG ROI demarcation in each subject, and clustering of the recording sites into two ROIs in the MNI coordinate space with little overlap. The TTS thus provided a reliable gross anatomical criterion for STG ROI parcellation.

Differences between high gamma activity elicited during listening and speaking were quantified as SIs at each STG recording site (**Figure 5C**). On the population level, significant suppression (*p* < 0.001, Wilcoxon signed rank tests) was observed in both STG ROIs, with no significant difference identified between the two ROIs (*p* = 0.63, Wilcoxon rank sum test). Instead, regions of suppression were interspersed with those exhibiting little-to-no suppression (cf. **Figure 4**). There appeared to be an overall lack of suppression between -20 and -40 mm on the *y*_mni_ axis when the data were pooled across subjects (white symbols, corresponding to -0.05 < SI < 0.05). However, most of those data points were contributed by the most posterior STG recording sites of subject R288 (hexagons). Therefore, the data should not be interpreted as suggesting that there is an orderly distribution of SIs along the long axis of the STG. This conclusion can only be made following a formal assessment of spatial distribution in the MNI coordinate space, which would require a larger number of subjects (see Nourski et al., 2014a) and is outside the scope of the current study.

**FIGURE 6:**
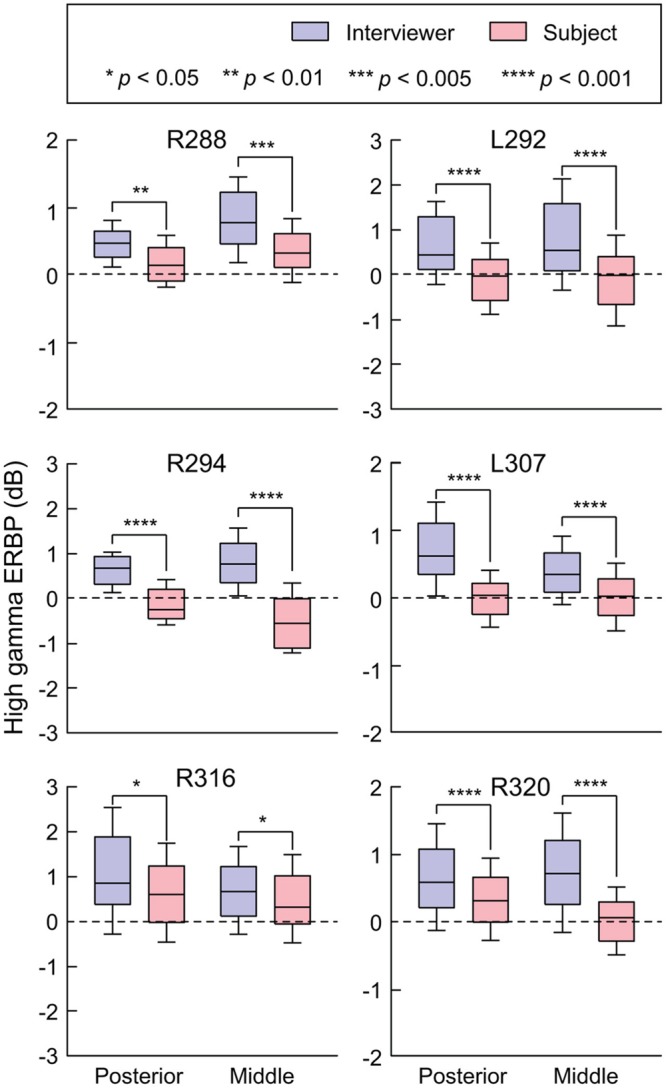
**Summary of STG ERBP analysis.** High gamma power was log-transformed and normalized to the mean power over the entire duration of the recording. Box plots show median, quartile, 10th and 90th percentile values of high gamma ERBP averaged over all interviewer’s and subject’s utterances (blue and red boxes, respectively) and recording sites within each ROI in each subject. Significance of ERBP differences was evaluated using Wilcoxon rank sum tests, followed by FDR correction.

As with examination of HG (see **Figure 3**), STG ROIs were further characterized using comparisons of high gamma activity normalized to the mean over the entire recording epoch (**Figure 6**). Significant suppression of high gamma activity during speaking was found in both posterior and middle STG in each subject. This suppression was further examined on a site-by-site basis in the three subjects with comprehensive lateral STG electrode coverage (L307, R288, and R320). In subject L307, 23 out of 26 STG sites (88.4%) exhibited significantly greater high gamma activity elicited during listening compared to speaking (Wilcoxon rank sum test, FDR-corrected, *p* < 0.05). No sites showed preference for self-vocalization. In subject R288, 12 out of 32 STG sites (37.5%) exhibited a significantly greater response when listening (*p* < 0.05), while two sites (6.25%) showed a reverse pattern, and 18 sites (56.25%) showed no difference. In subject R320, 15 out of 23 STG sites (65.2%) exhibited a significantly (*p* < 0.05) greater response when listening, while two sites (8.7%) showed a reverse pattern, and six sites (26.1%) showed no difference. Finally, there was no reliable difference between posterior and middle portions of lateral STG when comparing either responses elicited during listening or during speaking for all six subjects (*p* > 0.05).

### Modulation by Task

**FIGURE 7:**
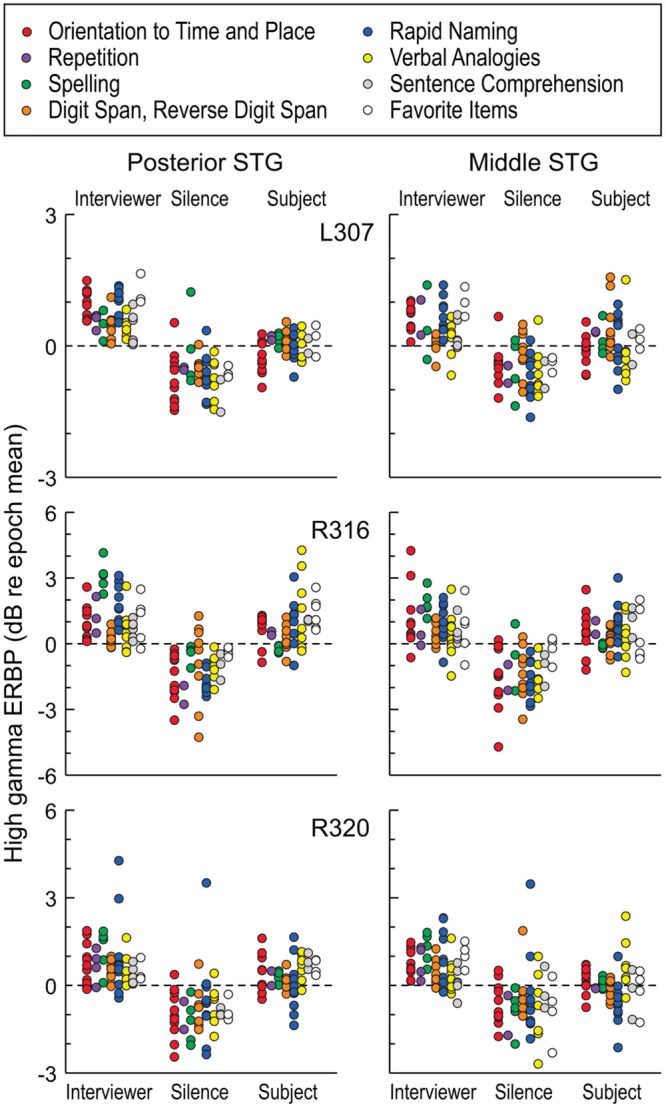
**High gamma ERBP measured in STG during interviewer’s and subject’s utterances and intervening silent intervals, segregated by task.** Different tasks of the expanded MMSE are denoted by different-colored circles. Each data point represents high gamma ERBP measured during one utterance, averaged over recording sites within a ROI (posterior and middle STG shown in left and right columns, respectively). Data are presented from the three subjects that underwent the expanded MMSE (top, middle, and bottom rows, respectively).

Modulation of high gamma activity on STG as a function of task can occur at a single site level, as exemplified by site ‘c’ in **Figure 4B**. At this site, activity during the Repetition task was suppressed when speaking relative to listening, yet was not suppressed during the Rapid Naming task. We further examined this property at a population level by exploring whether there were any systematic differences while listening and speaking as a function of specific tasks in the expanded MMSE. For this exploration, we included periods of silence between listening to questions and responding in order to account for activity related to either processing of the former or planning the latter. This analysis is illustrated in **Figure 7**. Although the low number of exemplars for each task within the dialog precluded a formal statistical assessment, it can be observed that no systematic task effects were apparent at the population level of STG. Periods of silence between questions and answers were typically associated with negative ERBP values, and, in general, responses while speaking were less than while listening. These findings indicate that the comparisons of high gamma activity while listening versus speaking, as depicted in **Figures 5C** and **6**, were not affected by systematic task-specific biases on the group (ROI) level. Given that individual sites on the STG can be modulated by task, these results may represent a “fine-grain” property that would not be seen at the ROI level. Acquisition of additional data would be required to systematically evaluate this property of the auditory cortex of the STG. At the ROI level, current observations provide a comparison point when examining higher cortical areas likely involved in the comprehension of questions, and the planning and execution of answers.

## Discussion

### Summary of Findings

Using a conversation-based paradigm modeled after a commonly used neurological screening tool for dementia (the MMSE), we examined high gamma ERBP at three stages of auditory cortical processing with regard to modulation when listening versus speaking. In posteromedial HG (core auditory cortex), no significant difference was found between activity during listening to the interviewer’s questions and the subject’s answers. This non-discriminate pattern changed within both anterolateral HG and lateral STG (non-core auditory cortical areas), where responses were significantly greater during listening compared to speaking. These observations are consistent with the idea that suppression of cortical activity to self-initiated speech is an emerging property of human non-core auditory cortex.

### Heschl’s Gyrus

This is the first detailed report to compare neural activity in human core auditory cortex during listening and speaking in a dialog-based paradigm. High gamma activity in posteromedial HG was not significantly modulated by speaker during the performance of the expanded MMSE. This observation is consistent with previous reports examining cortical high gamma activity in posteromedial HG, showing that this area responds indiscriminately to a wide array of simple and complex sounds, including intelligible and unintelligible speech (e.g., Brugge et al., 2009; Nourski et al., 2009a; Steinschneider et al., 2013) as well as while speaking or listening to playback of one’s own speech (Greenlee et al., 2014; Behroozmand et al., 2016). Further, high gamma activity in posteromedial HG is not strongly modulated by experimental context or specific task requirements (Steinschneider et al., 2014). Preliminary observations also demonstrate that early high gamma activity in posteromedial HG is even preserved under general anesthesia (Nourski et al., 2009b). In the setting of the current study, high gamma responses elicited by self-initiated vocalizations provide a further example of the breadth of acoustic inputs that activate core auditory cortex.

Auditory cortex in posteromedial HG exhibits phase locking to voice *F*_0_, particularly for male talkers whose speech is typically characterized by lower *F*_0_ values (e.g., Nourski and Brugge, 2011; Steinschneider et al., 2013; Behroozmand et al., 2016). These phase-locked responses would contribute to high gamma ERBP measured in posteromedial HG, and thus introduce a potential confound for comparisons between responses to utterances of different talkers with different *F*_0_s. Three out of six subjects in the present study (L292, R316, and R320) were female, and two of them (L292 and R316) had average *F*_0_ values higher than that of the male interviewer (see **Table 2**). Activity in posteromedial HG was not greater when listening to the interviewer compared to speaking in these subjects (see **Figures 2** and **3**). Further, the average voice *F*_0_ of the interviewer during these conversations (155 and 139.8 Hz) was at frequencies that were borderline with regard to the ability to elicit phase-locked responses (see Steinschneider et al., 2013; Behroozmand et al., 2016), again minimizing their potential contribution to our results.

It should be noted that the only subject where high gamma activity was significantly greater during speaking (L307) had the lowest voice *F*_0_ (120.7 Hz), and it was significantly lower than the interviewer’s voice *F*_0_. Even though phase-locked activity may have contributed to the observed significant difference in high gamma ERBP in this subject, it does not alter the conclusion that there is no systematic suppression of high gamma activity during self-generated speech at the level of posteromedial HG when compared to listening.

Utterances phrased as questions are often characterized by higher *F*_0_ values than utterances phrased as statements (e.g., Eady and Cooper, 1986). It’s not likely, however, that higher *F*_0_s associated with the interviewer’s questions would affect the results reported in the present study, as many of the interviewer’s utterances were phrased as statements (see Supplementary Table 1). Also, upward inflections in the *F*_0_ are often seen toward the end of a question, and do not substantially contribute to the overall high gamma response profiles when averaged over the entire utterance.

Given that responses when listening were greater than during self-generated speech in anterolateral HG and lateral STG, it is conceivable that these results could be skewed by the differences in voice *F*_0_s between the interviewer and the subjects. However, multiple studies have shown that these ROIs do not phase-lock to speech with voice *F*_0_s within the range occurring in the current study (e.g., Nourski and Brugge, 2011; Steinschneider et al., 2011; Steinschneider, 2013). This indicates that results represent genuine suppression of activity to self-initiated speech in these ROIs.

The finding that high gamma activity within posteromedial HG was not suppressed during self-vocalizations apparently contradicts human non-invasive studies. Neuromagnetic studies have revealed a decrement in the M100 component during speaking compared to listening (Houde et al., 2002; see also Numminen et al., 1999). However, the M100 is the sum of multiple generators with greater contributions from non-primary cortex on the superior temporal plane than HG (Scherg et al., 1989; Liégeois-Chauvel et al., 1994). Thus, the decrements seen while speaking could be a property of those non-primary areas rather than posteromedial HG.

In the marmoset, a New World monkey, two types of single-cell activity within primary and surrounding secondary auditory cortical areas have been described to occur during self-vocalization (Eliades and Wang, 2003). Vocalization-induced suppression of activity was seen in the majority of cells, but a significant minority showed increased discharges during self-vocalizations. Overall, summation of net activity generated by these cell populations was excitatory (Eliades and Wang, 2005). Our failure to find significant differences between responses during listening and speaking at the level of posteromedial HG may reflect limitations inherent to population responses (such as high gamma activity) in differentiating the fine-grain excitatory and inhibitory patterns associated with these two sources of acoustic inputs. On the other hand, mechanisms that preserve responses to self-vocalizations as seen in the current study at the level of core auditory cortex may be a necessary component of cortical pathways involved in self-monitoring of one’s own speech (Eliades and Wang, 2003, 2008; Rauschecker and Scott, 2009).

In contrast to posteromedial HG, high gamma activity within anterolateral portions of HG was both generally lower in magnitude and exhibited suppression during speaking. The decrement in response magnitude along HG has been a consistent finding in previous studies that examined high gamma activity using multiple sound stimuli in more controlled trial-based paradigms (e.g., Brugge et al., 2009; Nourski et al., 2009a; Nourski and Brugge, 2011). The change in magnitude of response along HG has been interpreted as reflecting a change from a core to a non-core field, and is consistent with anatomic parcellations of HG (e.g., Hackett et al., 2001). This interpretation is further supported by the transformation that occurs between posteromedial and anterolateral HG in terms of sensitivity to self-vocalization vs. listening as seen in the present study.

It is premature to draw conclusions regarding comparisons between the results obtained from HG in the only language-dominant hemisphere examined (subject L307) with those obtained from the five other subjects. Comparisons regarding response properties in HG (see **Figure 3**) require special caution because of the limited sampling in each subject. Thus, enhanced activity during speaking in posteromedial HG of subject L307 does not necessarily reflect a consistent difference in auditory processing between language dominant and non-dominant hemispheres at the level of auditory core cortex. What is consistent across all subjects, and which is a main finding of the present study, is that there is a lack of suppression of activity within auditory core cortex during speaking compared to listening regardless of the language dominance. Inclusion of many more subjects who clinically require placement of depth electrodes in the superior temporal plane of the language-dominant hemisphere would be required to reveal systematic differences across the hemispheres. It should also be noted that many models of speech perception posit that such differences emerge at later stages of auditory cortical processing (e.g., superior temporal sulcus; Leaver and Rauschecker, 2010).

### Superior Temporal Gyrus

The STG was strongly activated during our conversation-based paradigm in all subjects, including the five subjects in which the non-language dominant hemisphere was studied, as well as in the single subject (L307) with language dominant hemisphere electrode coverage. As previously reported by Greenlee et al. (2011), high gamma activity during speaking was generally attenuated when compared to listening to the playback of one’s own vocalizations. Suppressed activity during speaking occurred at sites in both posterior and middle portions of STG, which were intermingled with sites that exhibited no such suppression. This patchy distribution has been described in both humans and non-human primate models (Eliades and Wang, 2003; Greenlee et al., 2011). Interestingly, suppression of neural activity during self-vocalizations in the monkey was primarily seen in upper cortical laminae (Eliades and Wang, 2005). Activity generated within upper laminae would provide a major contribution to the population responses (high gamma) as captured by subdural electrodes immediately over lamina 1.

It is tempting to compare the overall magnitude of responses and the degree of self-vocalization suppression between anterolateral HG and STG. However, the extent of sampling was less for anterolateral HG and lateral STG responses were obtained from the pial surface as opposed to the brain parenchyma. For these reasons, we refrain from making conclusions regarding the relative degree of suppression of activity to self-vocalizations between anterolateral HG and STG.

### Phonetic Feature Representation

The lateral STG has been shown to encode phonetic features at both the single-electrode and population level (Mesgarani et al., 2014). The role of phonetic modulation in the neural activity within STG was not currently studied due to several technical restraints. First, the density of coverage over the posterior and middle STG in our subject cohort (between 5 and 32 recording sites) was considerably smaller than that in the study of Mesgarani et al. (2014), where the number of STG sites in each subject was generally greater than 80 and reached a maximum of 102. Next, the number of spoken sentences that was drawn upon for analysis of phonemic representation by Mesgarani et al. (2014) came from a well-designed acoustic-phonetic speech corpus (TIMIT; Garofolo, 1993) and greatly exceeded those in our data sets. Further, the conversational nature of the experimental paradigm in our study precluded the use of a local prestimulus baseline as utilized by Mesgarani et al. (2014). Finally, our study required participants to perform multiple verbal tasks while listening to the interviewer as opposed to passive listening to continuously presented sentences. It is possible that task demands might greatly increase the overall complexity of neural response patterns and thus partially mask effects based on phonetic representation. It should be stressed that our findings do not contradict the results of Mesgarani et al. (2014), but instead shed light on complementary organizational properties of the STG in an active conversation-based paradigm.

### Task Modulation

While at the population level of the STG, there was no systematic variation of high gamma activity according to task, activity at individual recording sites could show task-specific modulation during the subject’s verbal responses (see **Figure 4**). Modulation of high gamma activity at the level of the STG was not observed during the listening phase of the dialog. It is unclear what mechanisms drive this effect, and further work is clearly needed to categorize the functional specialization underlying task modulation observed at the level of single electrodes, and whether these effects occur in specific regions of posterior and middle STG.

## Conclusion

The utility of this conversation-based paradigm is supported by its ability to reliably reproduce findings such as speaker modulation on the lateral STG, and transformation of patterns of activity across regions of auditory cortex. It follows in the footsteps of previous intracranial studies demonstrating the ability to study social interactions, “cognitive ideas” and numerical processing in non-experimental settings (Derix et al., 2012, 2014; Dastjerdi et al., 2013). As such, this study lays the groundwork for analysis of this paradigm’s ability to rapidly evaluate task-specific activity related to language processing at higher levels of auditory-related cortex and its interface with regions of the brain involved in cognitive and affective functions. The expanded MMSE permits these examinations in a rapid and efficient manner, taking into account factors such as fatigue that commonly occur in patients being evaluated for their medically intractable epilepsy. While this study was limited to high gamma activity, it is recognized that future studies must also incorporate examination of lower frequency bands and coherence across sensory, cognitive, and affective areas. Finally, the results obtained from the expanded MMSE should permit formulation of novel hypotheses that can be tested using more formal, controlled experimental designs.

## Author Contributions

MS conceived the study; KN and MS designed the study; KN and AR collected the data; KN and MS analyzed and interpreted the data. All authors wrote the manuscript, approved its final version, and agreed to be accountable for all aspects of the work.

## Conflict of Interest Statement

The authors declare that the research was conducted in the absence of any commercial or financial relationships that could be construed as a potential conflict of interest.

## Abbreviations

ECoG: electrocorticography
ERBP: event-related band power
FDR: false discovery rate
HG: Heschl’s gyrus
MMSE: Mini-mental status exam
MNI: Montreal Neurological Institute
ROI: region of interest
SI: suppression index
STG: superior temporal gyrus
TTS: transverse temporal sulcus
