# Supplementary material for: Electrocorticographic Activation within Human Auditory Cortex during Dialog-Based Language and Cognitive Testing
Source: Front Hum Neurosci. 2016 May 4;10:202. doi: 10.3389/fnhum.2016.00202 (PMC4854871; doi:10.3389/fnhum.2016.00202)
Supplement: Supplementary file 1 [file Data_Sheet_1.DOCX]

Supplementary Material

Electrocorticographic activation within human auditory cortex during dialogue-based language and cognitive testing

Kirill V. Nourski, Mitchell Steinschneider*, Ariane E. Rhone

*** Correspondence:** Mitchell Steinschneider: mitchell.steinschneider@einstein.yu.edu

# Supplementary Figures and Tables

## Supplementary Figures


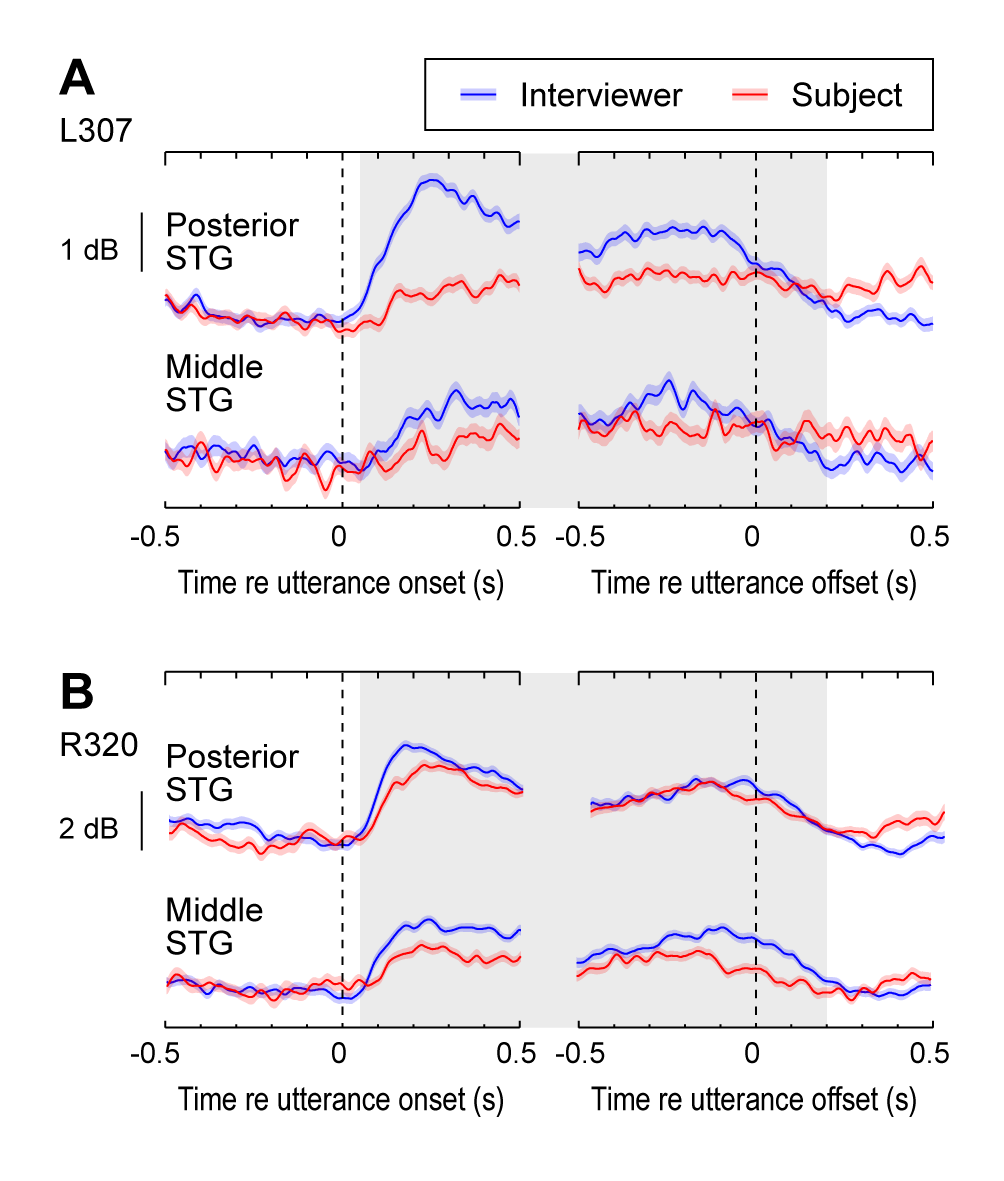


**Supplementary Figure 1.** Time course of high gamma ERBP at utterance onset and offset (left and right panels, respectively). Data from two subjects L307 and R320 (A and B, respectively). High gamma ERBP was averaged across posterior and middle STG recording sites, and across all interviewer’s and subject’s utterances. Shaded areas of each plot represent standard error of the mean. Analysis window used for utterance-by-utterance analysis of high gamma ERBP (50 ms after onset to 200 ms after offset) is highlighted in gray.

## Supplementary Tables

**Supplementary Table 1. Mini-Mental Status Examination (modified after Folstein et al., 1975).**

Questions in *italics* are part of the extended questionnaire used in subjects L307, R316 and R320.

| **Orientation to Time** | | **Correct** | | **Incorrect** | | |  | | | |
| --- | --- | --- | --- | --- | --- | --- | --- | --- | --- | --- |
| What is the year? | | **□** | | **□** | | |  | | | |
| What season is it? | | **□** | | **□** | | |  | | | |
| What is the month? | | **□** | | **□** | | |  | | | |
| What is today’s date? | | **□** | | **□** | | |  | | | |
| What is the day of the week today? | | **□** | | **□** | | | Total: ______ | | | |
| *What is your favorite food?* | |  | |  | | |  | | | |
|  | |  | |  | | |  | | | |
| **Orientation to Place** | |  | |  | | |  | | | |
| What country are we in? | | **□** | | **□** | | |  | | | |
| What state are we in? | | **□** | | **□** | | |  | | | |
| What city are we in? | | **□** | | **□** | | |  | | | |
| What is this place? | | **□** | | **□** | | |  | | | |
| What room is this? | | **□** | | **□** | | | Total: ______ | | | |
| *What is your favorite TV show?* | |  | |  | | |  | | | |
| **Immediate recall** | |  | |  | | |  | | | |
| May I test your memory? I’m going to give you three words to remember. The words are: Ball… flag… tree.  What did I say? | | | | | | | | | | |
| Ball | | **□** | | **□** | | |  | | | |
| Flag | | **□** | | **□** | | |  | | | |
| Tree | | **□** | | **□** | | | Total: ______ | | | |
| *What is your favorite movie?* | |  | |  | | |  | | | |
|  | |  | |  | | |  | | | |
| **Calculation and attention [use the higher score]** | | | | | | | | | | |
| Please count backwards by three beginning with 100 [stop after 5 subtractions]. | | | | | | | | | | |
| 97 | | **□** | | **□** | | |  | | | |
| 94 | | **□** | | **□** | | |  | | | |
| 91 | | **□** | | **□** | | |  | | | |
| 88 | | **□** | | **□** | | |  | | | |
| 85 | | **□** | | **□** | | |  | | | |
| Please spell the word “WORLD” backwards. | | | | | | | | | | |
| D | | **□** | | **□** | | |  | | | |
| L | | **□** | | **□** | | |  | | | |
| R | | **□** | | **□** | | |  | | | |
| O | | **□** | | **□** | | |  | | | |
| W | | **□** | | **□** | | | Total: ______ | | | |
| *What is your favorite color?* | |  | |  | | |  | | | |
| **Delayed verbal recall** | |  | |  | | |  | | | |
| Please recall the three words I previously asked you to remember. | | | | | | | | | | |
| Ball | | **□** | | **□** | | |  | | | |
| Flag | | **□** | | **□** | | |  | | | |
| Tree | | **□** | | **□** | | | Total: ______ | | | |
|  | |  | |  | |  | | |  |  |
| **Naming** |  | |  | |  | | | | |  |
| What is this? [show wristwatch] | | **□** | | **□** | | |  | | | |
| What is this? [show pen] | | **□** | | **□** | | | Total: ______ | | | |
|  | |  | |  | | |  | | |  |
| **Repetition** | |  | |  | | |  | | |  |
| Please repeat the following: | | | | | | | | | |  |
| “No ifs, ands or buts”. | | **□** | | **□** | | | Total: ______ | | |  |
|  | |  | |  | | |  | | |  |
| **3-Stage Command** | |  | |  | | |  | | |  |
| Please take the paper in your hand, fold it in half and put it next to you. | | | | | | | | | |  |
| [Takes] | | □ | | □ | | |  | | |  |
| [Folds] | | **□** | | **□** | | |  | | |  |
| [Puts] | | **□** | | **□** | | | Total: ______ | | |  |
|  | |  | |  | | | MMSE total: ______ | | |  |
|  | |  | |  | | |  | | |  |
| ***Fund of knowledge*** | | | | | | | | | |  |
| *Please name ten animals that live in the zoo.* | | | | | | | | | |  |
| *Please name ten animals that live on a farm.* | | | | | | | | | |  |
| *Please name ten words that describe things you do every day, such as “eat”.* | | | | | | | | | |  |
| *What is celebrated on the 4th of July?* | | | | | | | | | |  |
| *Who wrote “Romeo and Juliet”?* | | | | | | | | | |  |
| *Why does oil float on water?* | | | | | | | | | |  |
| *What does the stomach do?* | | | | | | | | | |  |
| *What is the color of rubies?* | | | | | | | | | |  |
|  | | | | | | | | | |  |
| ***Verbal analogies*** | | | | | | | | | |  |
| *I’ll start a sentence, and you finish it with one word.* | | | | | | | | | |  |
| *A pickle is fat, a pencil is…?* | | | | | | | | | |  |
| *A bee has a hive, a man has a…?* | | | | | | | | | |  |
| *Trees have bark, people have…?* | | | | | | | | | |  |
| *A rabbit has a tail, a train has a…?* | | | | | | | | | |  |
| *A foot has inches, a minute has…?* | | | | | | | | | |  |
| *Ice is solid, water is…?* | | | | | | | | | |  |
| *Trees have sap, animals have…?* | | | | | | | | | |  |
|  | | | | | | | | | |  |
| ***Spelling*** | | | | | | | | | |  |
| *Please spell the following words:* | | | | | | | | | |  |
| *Watch* | | | | | | | | | |  |
| *Circle* | | | | | | | | | |  |
| *Kitchen* | | | | | | | | | |  |
| *Educate* | | | | | | | | | |  |
| *Surprise* | | | | | | | | | |  |
| ***Sentence comprehension*** | | | | | | | | | |  |
| *Please listen to the following sentences and answer the questions:* | | | | | | | | | |  |
| *Sally will play softball again when she’s better. Missing practice is not so bad because she has a lot of work to do. Why isn’t Sally playing softball?* | | | | | | | | | |  |
| *Mary and Suzie love to play with each other every day after school. But each night just before they go to bed they keep arguing and fighting. How do Mary and Suzie know each other?* | | | | | | | | | |  |
| *Julie thinks studying for tests is boring. She would much rather play soccer or go to a movie. Of course, she needs to get ready for tomorrow. What’s happening tomorrow?* | | | | | | | | | |  |
|  | | | | | | | | | |  |
| ***Orientation to Time*** | | | | | | | | | |  |
| *Tell me the days of the week backwards, starting with Thursday.* | | | | | | | | | |  |
| *What month comes right before March?* | | | | | | | | | |  |
| *During the race, a runner finished at 11:30. Another finished 15 minutes earlier. What time did the faster runner finish?* | | | | | | | | | |  |
|  | | | | | | | | | |  |
| ***Digit span*** | | | | | | | | | |  |
| *I am going to give you some numbers. Listen and repeat them back to me:* | | | | | | | | | |  |
| *9 6 1 5* | | | | | | | | | |  |
| *3 8 7 2* | | | | | | | | | |  |
| *7 1 6 4 3* | | | | | | | | | |  |
| *9 2 5 8 4* | | | | | | | | | |  |
| *3 6 5 2 4 1* | | | | | | | | | |  |
| *8 3 1 7 9 6* | | | | | | | | | |  |
|  | | | | | | | | | |  |
| ***Reverse digit span*** | | | | | | | | | |  |
| *I am going to give you some numbers. Listen and repeat them back to me backwards. So, if I say 1 2, you say 2 1.* | | | | | | | | | |  |
| *4 7 2* | | | | | | | | | |  |
| *9 3 8* | | | | | | | | | |  |
| *5 4 8 1* | | | | | | | | | |  |
| *6 2 9 7* | | | | | | | | | |  |
|  | | | | | | | | | |  |
| ***Rhyming*** | | | | | | | | | |  |
| *Tell me five words that rhyme with “make”* | | | | | | | | | |  |
| *Tell me five words that rhyme with “ball”* | | | | | | | | | |  |
|  | | | | | | | | | |  |
| ***Naming*** | | | | | | | |  |  |  |
| *Please answer as quickly as possible. Tell me:* | | | | | | | |  |  |  |
| *Part of a fish* | | | | | | | |  |  |  |
| *Something square* | | | | | | | |  |  |  |
| *Part of a book* | | | | | | | |  |  |  |
| *Something you catch* | | | | | | | |  |  |  |
| *Something metal* | | | | | | | |  |  |  |
| *A green vegetable* | | | | | | | |  |  |  |
| *Something white* | | | | | | | |  |  |  |
| *Something slippery* | | | | | | | |  |  |  |
| *A coin* | | | | | | | |  |  |  |
| *A salty food* | | | | | | | |  |  |  |
